# Supplementary material for: Transcriptomic Analysis of the Effect of Pruning on Growth, Quality, and Yield of Wuyi Rock Tea
Source: Plants (Basel). 2023 Oct 20;12(20):3625. doi: 10.3390/plants12203625 (PMC10610282; doi:10.3390/plants12203625)
Supplement: Supplementary file 1 [file plants-12-03625-s001.zip › plants-2573601-supplementary.pdf]

Supplementary Materials

Table S1 Overview of transcriptomic sequencing in tea leaves

Sample	Raw Reads	Raw bases	Clean Reads	Clean Base(G)	Error Rate(%)
MC-1	43512646	6.52	42172184	6.33	0.03
MC-2	41245880	6.18	40066860	6.01	0.03
MC-3	43004222	6.45	41740838	6.26	0.03
MP-1	38908256	5.83	37648946	5.65	0.03
MP-2	40024568	6.00	39034110	5.86	0.03
MP-3	44819000	6.72	43317594	6.50	0.03
Total/Average	251514572	6.28	243980532	6.10	0.03

Note: MC: Tea tree unpruned; MP: Tea tree pruned

Table S2 Efficiency statistics of transcriptome data compared with reference genomes

Sample	Total reads	Total mapped	Multiple mapped	Uniquely mapped	Read-1 mapped	Read-2 mapped	Reads map to '+'	Reads map to '-'
MC-1	42172184	39770212 (94.3%)	2985238 (7.08%)	36784974 (87.23%)	18392487 (43.61%)	18392487 (43.61%)	18392487 (43.61%)	18392487 (43.61%)
MC-2	40066860	37828644 (94.41%)	2856432 (7.13%)	34972212 (87.28%)	17486106 (43.64%)	17486106 (43.64%)	17486106 (43.64%)	17486106 (43.64%)
MC-3	41740838	39367906 (94.32%)	2996280 (7.18%)	36371626 (87.14%)	18185813 (43.57%)	18185813 (43.57%)	18185813 (43.57%)	18185813 (43.57%)
MP-1	37648946	35519428 (94.34%)	2681390 (7.12%)	32838038 (87.22%)	16419019 (43.61%)	16419019 (43.61%)	16419019 (43.61%)	16419019 (43.61%)
MP-2	39034110	36804738 (94.29%)	2656414 (6.81%)	34148324 (87.48%)	17074162 (43.74%)	17074162 (43.74%)	17074162 (43.74%)	17074162 (43.74%)
MP-3	43317594	40750932 (94.07%)	3029678 (6.99%)	37721254 (87.08%)	18860627 (43.54%)	18860627 (43.54%)	18860627 (43.54%)	18860627 (43.54%)

Note: MC: Tea plant unpruned; MP: Tea plant pruned; The reference genome was GCF_004153795.1_AHAU_CSS_1_genomic.fna.gz. Download address: https://ftp.ncbi.nlm.nih.gov/genomes/all/GCF/004/153/795/GCF_004153795.1_AHAU_CSS_1/; **Total Reads**: Total number of clean reads; **Total mapped**: The number of reads compared to the reference genome; **Multi mapped**: The number of reads multiple compared to the reference genome; **Unique mapped**: The number of reads only compared to the reference genome; **Read-1 mapped**: Number of successes comparison in read1; **Read-2 mapped**: Number of successes comparison in read2; **Reads map to '+'**: Number of reads successfully comparison on the positive chain of the genome; **Reads map to '-':** Number of reads successfully comparison on the negative chain of the genome.

Table S3 Analysis of the growth and quality indicator of tea plant

	Index	MC	MP	Adjusted *p*-value
Growth index	Leaf area (cm^2)	21.64±0.48	28.77±0.30	< 0.05
	Hundred-bud weight (g）	107.90±2.23	151.70±5.71	< 0.05
	ChlorophyII content (SPAD)	35.37±0.32	38.10±0.69	< 0.05
	Yield (kg/ha)	189.33±4.04	350.00±3.00	< 0.05
Quality index	Tea polyphenol (mg/g)	15.84±0.36	16.33±0.42	0.19
	Theanine (mg/g)	1.29± 0.02	1.31±0.02	0.35
	Caffeine (mg/g)	2.99± 0.03	2.78±0.05	< 0.05
	Flavone (mg/g)	1.11± 0.01	0.97±0.02	< 0.05
	Free amino acid (mg/g)	4.25± 0.05	2.96±0.12	< 0.05
	Soluble sugar (mg/g)	3.46± 0.02	3.66±0.04	< 0.05

Note: MC: Tea plant unpruned; MP: Tea plant pruned.

Table S4 The basic conditions of tea plantation during the experiment

	Maximum temperature (℃)	Minimum temperature (℃)	Mean temperature (℃)	Number of sunny days (Day)	Precipitation days (Day)	Amount of precipitation (mm)	Relative humidity (%)	Pest and disease control
August 2021	35	23	27.7	13	18	194.5	79	The pest and disease control in tea plantation was based on organic pesticides, the main organic pesticides used were matrine and azadirachtin, each organic pesticide was used three times a year in March, September and October, two organic pesticides were used alternately, and the use time interval of 10 days.
September 2021	36	20	27.4	21	9	13.6	71	
October 2021	33	11	20.9	17	14	35.6	73	
November 2021	23	3	14	18	12	77.3	76	
December 2021	20	2	10	22	9	32.7	71	
January 2022	20	1	9.7	14	17	54.2	80	
February 2022	23	1	7.6	10	18	151	82	
March 2022	27	3	16.1	13	18	309.9	79	
April 2022	33	6	18	13	17	112.6	74	
